# Editorial: Megakaryocytes as regulators of tumor microenvironments

**DOI:** 10.3389/fonc.2022.1090658

**Published:** 2022-11-25

**Authors:** Anna Rita Migliaccio, Alessandra Balduini, Huichun Zhan

**Affiliations:** ^1^ Center for Integrated Biomedical Research, Campus Bio-medico, Rome, Italy; ^2^ Altius Institute for Biomedical Sciences, Seattle, WA, United States; ^3^ Department of Molecular Medicine, University of Pavia, Pavia, Italy; ^4^ Department of Biomedical Engineering, Tufts University, Medford, MA, United States; ^5^ Department of Medicine, Stony Brook School of Medicine, Stony Brook, NY, United States; ^6^ Medical Service, Northport VA Medical Center, Northport, NY, United States

**Keywords:** megakaryocytes, microenvironment, myelofibrosis, proinflammatory cytokines, tumor progression

## Introduction

This Research Topic contains eight articles dedicated to summarizing recent knowledge on the functions of megakaryocytes as regulators of the microenvironment, how these functions are altered in Philadelphia-negative myeloproliferative neoplasms (MPN), and how they may be targeted for therapy (Khatib-Massalha et al., Varricchio and Hoffman, Zhan and Kaushansky, Karagianni et al., Malara et al., Pozzi et al., Verachi et al., Eaton et al.). The “theme” was inspired by recent paradigm shifts in several scientific areas including the identification of subclasses of megakaryocytes with functions other than producing platelets, the role played by the microenvironment in cancer progression and played by the malignant megakaryocytes in inducing a malignant hematopoietic stem cell (HSC)-supportive microenvironment.

## Novel subclasses of megakaryocytes

Recent single cell expression profiling of megakaryocytes purified from human and mouse embryos, as well as from adult bone marrow (BM) and lung, identified that, in addition to megakaryocytes responsible for producing platelets, there are at least three populations, each one exerting a specific function in development and adult homeostasis (1–5). These populations are niche-poised megakaryocytes, which arise from dedicated endothelial cells derived from the hemogenic endothelium of the embryonic aorta-gonad-mesonephro region and are responsible for producing the extracellular matrix during the process of organogenesis; Immune-poised megakaryocytes, which are produced in response to inflammatory signals and participate to the host defense against infective agents. HSC-supporting megakaryocytes which are present in the BM in close proximity to the vascular niche and support the HSC by producing PF4 and TGF-β. The Frenette and Li laboratories provided the first evidence for the existence of HSC-supportive megakaryocytes (6, 7). Dr. Simon Mendez-Ferrer wrote the perspective summarizing the novel megakaryocyte subpopulations and functions and the evidence suggesting that normal megakaryocytes regulate the functions of the HSC while their malignant counterparts induce the microenvironment dysfunctions which sustain progression of hematologic malignancies, including MPN(Khatib-Massalha et al.).

## Microenvironment and cancer progression

It is widely recognized that driver mutations play a double sword rule during malignant transformation (8): they alter the proliferation of normal cells transforming them into tumor cells and induce tumor cells to produce pro-inflammatory cytokines, and other factors, which turn the “normal” microenvironment into a “tumor”-supporting microenvironment (9). This concept is guiding the development of strategies that treat cancer by combining drugs targeting the driver mutations with those inhibiting the supporting microenvironment. In MPN, the driver mutations induce hyperproliferation of the HSC that manifest itself with different phenotypes: increased production of erythroid cells, resulting in polycythemia vera, or of megakaryocytes (10). Megakaryocyte hyperproliferation may then be associated with increased terminal maturation, resulting in essential thrombocythemia, or with a block of maturation, resulting in pre-myelofibrosis. These relatively benign diseases can be controlled with minimal therapeutic intervention for many years. However, an event secondary to the driver mutation leads MPN patients toward developing myelofibrosis and to a fatal outcome within a few years (10). Myelofibrosis is an unmet clinical need since the therapeutic interventions currently available are effective in curing its symptoms but do not halt its progression toward the fatal outcome (11). Dr. Hoffman, who pioneered the studies indicating that in myelofibrosis the malignant megakaryocytes induce the formation of a “malignant” HSC-supporting microenvironment by secreting abnormally high levels of TGF-β (12), reviews recent knowledge on the pro-inflammatory signals produced by the malignant megakaryocytes which turn the microenvironment into a malignant HSC-supportive microenvironment that are currently under clinical investigation for microenvironment targeted therapy in myelofibrosis (Varricchio and Hoffman).

## Malignant megakaryocytes as shapers of the hematopoietic niches

In the BM, the HSC are in close contacts with the endothelial cells, the vascular HSC-niche, which produce factors (SCF, SDF1, and others) necessary for their retention and proliferation (13). The increased angiogenesis observed in myelofibrosis suggest that the endothelial niche is altered in this disease (10). Zhan and Kaushansky have been among the first to prove that the *JAK2* mutation alters the HSC-supporting ability of endothelial cells in myelofibrosis (14). Here, they summarize how megakaryocytes and endothelial cells cooperate in regulating HSC functions and how disruption of their interplay contributes to expanding the malignant HSC clone in MPN (Zhan and Kaushansky).

The numerous bone spicula found in the BM from myelofibrosis patients indicate that the osteoblasts, an additional component of the BM microenvironment (13), are also altered in myelofibrosis. As recently reviewed by Dr. Ravid (15), osteoblast alterations contribute to the osteosclerosis that significantly increases the risk of bone fractures in male patients, where risk calculations are not confounded by changes occurring in sex hormones in age-matched females with MPN. Here, Dr Karagianni et al. provide mechanistic insights into how in male MPN patients the malignant megakaryocytes are responsible to induce the formation of weaker bones by activating osteoblast proliferation while inhibiting their maturation into calcium-secreting cells.

## Megakaryocyte and CD34+-biomarkers for monitoring disease progression in myelofibrosis

Since BM biopsy is a cumbersome non-patient friendly procedure, biomarkers detectable in blood are badly needed to follow disease progression and make risk stratification assessments in myelofibrosis. The Balduini laboratory, who first discovered that great numbers of megakaryocytes from the BM of myelofibrosis patients produce collagens, fibronectin and other extracellular matrix components (16, 17), describes here that the plasma levels of EDA fibronectin, possibly produced by the malignant megakaryocytes, are elevated and predict high allele burden of *JAK2V617F* and splenomegaly progression in myelofibrosis (Malara et al.).

In addition to megakaryocyte abnormalities, alterations induced by the driver mutations in the HSC/progenitor cell compartments contribute to the insurgency of myelofibrosis (10). Dr Masselli describes here that the malignant HSC express increased levels of CCR2 (C-C chemokine receptor 2, the ligand for monocyte chemoattractant protein-1) (18) that correlate with the severity of fibrosis in MPN, suggesting that the levels of CCR2 expressed by the circulating malignant CC34+ cells represent a biomarker to track disease progression in these patients (Pozzi et al.).

## Megakaryocyte abnormalities as micro-environment targeted therapy for myelofibrosis

The ultimate goal of studies on the alterations of the malignant megakaryocytes is to identify druggable targets for to cure myelofibrosis (19). The malignant megakaryocytes produce high levels of CXCL8 (interleukin 8) (20) and its plasma levels predict poor outcomes in myelofibrosis patients (21). The Migliaccio laboratory demonstrates here that the megakaryocytes from the *Gata1^low^* mouse model of myelofibrosis express high levels of CXCL1, the murine equivalent of CXCL8, and that treatment with an inhibitor of its receptors CXCR1/R2, Reparixin, reduces fibrosis and reactivates BM hematopoiesis in this mouse model (Verachi et al.), providing the preclinical rationale for a clinical trial that will evaluate Reparexin for the treatment of myelofibrosis under investigation (MPN-RC 120, NCT pending).

## How the same mutations may induce MPN with different phenotypes?

Although *TPO* is a growth factor mostly controlling thrombocytopoiesis (22), mutations in its receptor *MPL* are found not only in patients with essential thrombocythemia but also in patients with polycythemia vera (10). Using an elegant genetic approach, Dr. Falet provides here a clue to explain why *MPL* mutations may manifest themselves with either an erythroid or megakaryocyte phenotype by showing that endocytosis of MPL regulates both megakaryocyte and erythroid maturation in mice (Eaton et al.).

## Conclusions

The papers in this topic do not intend to provide a final answer on the role of megakaryocytes in health and disease. However, they provide an overview of the areas currently most actively investigated and predict the studies that will be published soon on this Research Topic.

## Author contributions

All authors listed have made a substantial, direct, and intellectual contribution to the work and approved it for publication.

## Funding

This study was supported by grants from the National Cancer Institute (P01-CA108671), the National Heart, Lung and Blood Institute (1R01-HL134684), Associazione Italiana Ricerca Cancro (AIRC, IG23525) (AM), (AIRC, IG18700) (AB), NIH R01 HL134970 and VA Merit Award BX003947 (HZ).

## Conflict of interest

The authors declare that the research was conducted in the absence of any commercial or financial relationships that could be construed as a potential conflict of interest.

## Publisher’s note

All claims expressed in this article are solely those of the authors and do not necessarily represent those of their affiliated organizations, or those of the publisher, the editors and the reviewers. Any product that may be evaluated in this article, or claim that may be made by its manufacturer, is not guaranteed or endorsed by the publisher.
